# Braincase simplification and the origin of lissamphibians

**DOI:** 10.1371/journal.pone.0213694

**Published:** 2019-03-22

**Authors:** Jade B. Atkins, Robert R. Reisz, Hillary C. Maddin

**Affiliations:** 1 Department of Earth Sciences, Carleton University, Ottawa, ON, Canada; 2 Department of Biology, University of Toronto at Mississauga, Mississauga, ON, Canada; 3 International Center of Future Science, Dinosaur Evolution Research Center, Jilin University, Changchun, China; Natural History Museum Luxembourg, LUXEMBOURG

## Abstract

Dissorophoidea, a group of temnospondyl tetrapods that first appear in the Late Carboniferous, is made up of two clades ⎼ Olsoniformes and Amphibamiformes (Branchiosauridae and Amphibamidae) ⎼ the latter of which is widely thought to have given rise to living amphibians (i.e., Lissamphibia). The lissamphibian braincase has a highly derived morphology with several secondarily lost elements; however, these losses have never been incorporated into phylogenetic analyses and thus the timing and nature of these evolutionary events remain unknown. Hindering research into this problem has been the lack of phylogenetic analyses of Dissorophoidea that includes both taxonomically dense sampling and specific characters to document changes in the braincase in the lineage leading to Lissamphibia. Here we build on a recent, broadly sampled dissorophoid phylogenetic analysis to visualize key events in the evolution of the lissamphibian braincase. Our ancestral character state reconstructions show a clear, step-wise trend towards reduction of braincase ossification leading to lissamphibians, including reduction of the sphenethmoid, loss of the basioccipital at the Amphibamiformes node, and further loss of both the basisphenoid and the hypoglossal nerve foramina at the Lissamphibia node. Our analysis confirms that the highly derived condition of the lissamphibian braincase is characterized by overall simplification in terms of the number and extent of chondrocranial ossifications.

## Introduction

The distinctive morphology of living lissamphibians (frogs, salamanders, and caecilians) has been a leading cause in the lack of resolution of both their relationships to one another, and from which group of extinct tetrapods they are derived. Different phylogenetic analyses have placed them entirely within Temnospondyli [1–7], entirely within Lepospondyli [8–10], a group that may be polyphyletic [11], or a combination thereof, where Batrachia (frogs and salamanders) reside within Temnospondyli and Apoda (caecilians) reside within Lepospondyli [12–15]. Despite all three hypotheses, and even a recent fourth that recovers Batrachia within Dissorophoidea and Apoda within Stereospondyli [16], maintaining a presence in the literature, there has been a growing consensus that lissamphibians are a monophyletic assemblage derived from within Temnospondyli [4,7,17–22], and more specifically from within the amphibamid dissorophoids [3,6–7,21–24].

This phylogenetic framework permits investigations of patterns of morphological evolution of lissamphibians, including the origin of their highly derived form. A combination of new discoveries [16,25–26] as well as extensive reanalysis of existing material [21,27–32] provide the foundation for potentially high-resolution investigations of morphological evolution with great numbers of taxa and forms. Unfortunately, many currently available phylogenetic analyses take place at either very broad taxonomic levels [13,21,33–34], or very fine, taxonomically exclusive, levels [19,35–36]. In the case of the former, detailed variation is glossed over by pruning of many taxa to cover a broader sampling; whereas with the latter, the distribution of traits at higher clade levels is missed due to entire clades being excluded.

**Fig 1:**
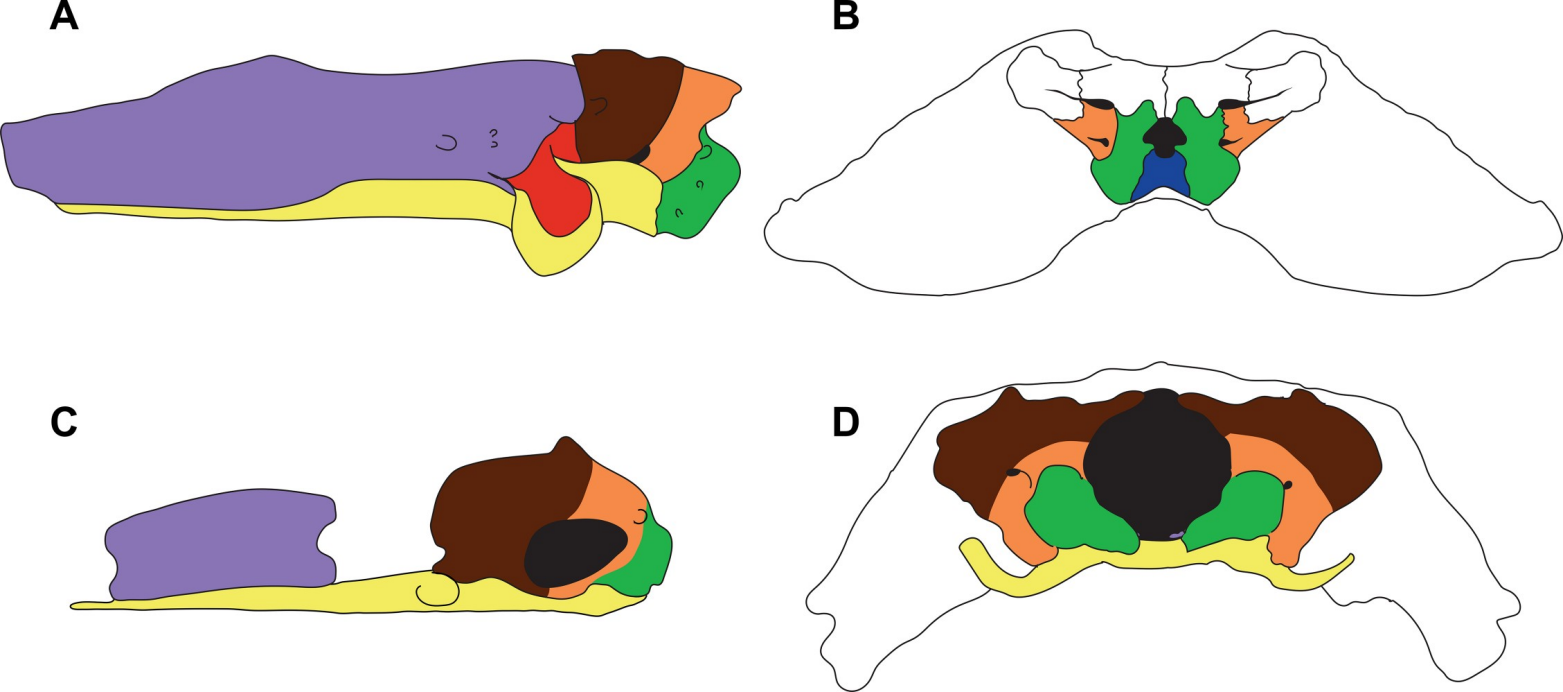
**A comparison between the braincases of the extinct tetrapod *Eryops* (A, B) and the extant salamander *Hynobius* (C, D), in lateral (A, C) and occipital (B, D) views, summarizing the main differences between lissamphibian braincase composition and morphology and that of other tetrapods.** The sphenethmoid is purple, the basioccipital is blue, the basisphenoid is red, the exoccipitals are green, the parasphenoid is yellow, the opisthotic is orange and the prootic is brown.

One such series of evolutionary events concerns the origin of the relatively simple condition, in terms of the number of ossifications and the extent of ossification of individual bones, of the braincase in lissamphibians in comparison to that of amniotes and to other temnospondyls. Extant amniotes and extinct temnospondyls (e.g. *Edops*, *Eryops*) have numerous ossifications making up the braincase, including the parasphenoid (membrane bone often considered part of the braincase), sphenethmoid, basisphenoid, prootics, opisthotics, paired exoccipitals and median basioccipital (Fig 1) [37–44]. The presence of these braincase elements is considered to represent the ancestral condition for tetrapods [42]; however, the presence and degree of ossification in these elements varies in temnospondyls, with basal members such as *Edops* having a heavily ossified braincase compared to more derived members such as *Eryops* [43–44]. Both stereospondyls and dissorophoids vary in the number and degree of ossifications in the braincase, with many more derived stereospondyls lacking an ossified basioccipital (e.g. *Gerrothorax*) or possessing a weakly ossified basisphenoid (e.g. *Mastodonsaurus*) when compared to basal stereospondyls (e.g. *Archegosaurus*) [45–47]. In the dissorophoid lineage, some members appear to possess an almost entirely cartilaginous braincase (e.g. branchiosaurids, and micromelerpetids) [48–49].

Extant lissamphibians reduce the braincase even further compared to all other temnospondyls as they possess only the parasphenoid, a reduced sphenethmoid, the otic elements and paired exoccipitals as discrete ossifications (Fig 1) [6,37–42]. In the case of the basisphenoid and basioccipital, the cartilaginous precursors are considered absent from the chondrocranium of lissamphibians [7,37,40,42,50] (but see the Discussion). Additionally, other bones that are commonly found in tetrapods are comparatively reduced (e.g. the sphenethmoid) [37,42] or absent (e.g. supraoccipital [6,50]) in lissamphibians (Fig 1). In the case of the supraoccipital, caecilian lissamphibians further lack its cartilage precursor, the tectum synoticum [37]. The condition of the lissamphibian braincase has historically led some authors to propose it to represent the ancestral tetrapod condition [51–53]; however, other authors have considered it to represent a derived condition [37,54–55]. Regardless, the timing and nature of morphological evolutionary events leading to the origin of the reduced lissamphibian condition remain unclear.

The current study attempts to resolve these issues by utilizing a broadly sampled phylogeny for Dissorophoidea such that details and patterns of braincase evolution in this group, and by proxy lissamphibians, can be ascertained. Here we built on the recently published Dissorophoidea matrix by Schoch [7] and sampled 42 dissorophoid taxa (four from Micromelerpetidae, 17 from Olsoniformes, and 21 from Amphibamiformes). The original matrix by Schoch [7] included 28 dissorophoid taxa (four from Micromelerpetidae, eight from Olsoniformes, and 16 from Amphibamiformes). Our sampling is as inclusive as possible and represents an improvement in this regard to earlier analyses. For example, Schoch [56] included 25 extinct dissorophoid taxa, and Fröbisch and Schoch [19] included 31 extinct dissorophoid taxa. Maddin *et al*. [21] is the only recent analysis to broadly sample both extinct and extant members of Lissamphibia, but even this matrix only included 27 dissorophoid taxa, of which 10 were caecilian taxa. Our analysis and the resulting trees allow us to reconstruct the ancestral character states and present a hypothesis of braincase evolution in the lineage leading to Lissamphibia. We discuss these results as they relate to the origin of the lissamphibian form.

## Methods

### Phylogenetic analysis

We approached this problem from the perspective that lissamphibians are monophyletic dissorophoid temnospondyls, which is made up of two clades: the Olsoniformes, and the newly proposed Amphibamiformes (Amphibamidae and Branchiosauridae [7]). To produce a broadly sampled phylogeny for Dissorophoidea, we utilized the latest published matrix from Schoch [7]. To this matrix we added three additional braincase characters to capture variation in the presence or absence of braincase bones (S1 Appendix, characters 109–111). We took the decision to include as many taxa as possible in order to avoid subjective user bias in the exclusion of taxa that might actually be important. As such, twelve extinct taxa were also added to the matrix: Eryopidae, *Tambachia*, *Anconastes*, Admiral and Rio Arriba taxa, *Kamacops*, *Brevidorsum*, *Reiszerpeton*, *Aspidosaurus*, *Platyhystrix*, *Rubeostratilia*, and *Plemmyradytes*. Additionally, three extant taxa were added to the matrix: representatives for each of the extant lissamphibian groups (*Ascaphus* for frogs, *Hynobius* for salamanders, and *Rhinatrema* for caecilians). The final matrix thus consisted of 48 taxa, including the outgroup taxon, *Dendrysekos* (= *Dendrerpeton* [44]) and 111 characters. Taxa were coded for characters not present in their original matrix from the literature and some first-hand observations of specimens (see S2 Appendix for a full list of taxa and sources).

The final matrix was analyzed in PAUP using maximum parsimony and in MrBayes using Bayesian Inference. The PAUP analysis was run using version 4.0a161 [57] for MacIntosh. *Dendrysekos* was set as the outgroup for rooting. The heuristic search option and tree bisection-reconnection branch-swapping algorithm were used with 10 000 additional random sequence replicates selected. Multistate taxa were treated as polymorphic, and all characters were unordered and weighted equally. The Bayesian analysis was run in MrBayes version 3.1.2 [58] using the default Mk model and running a Markov chain Monte Carlo for 5 million generations, with a sampling frequency of 100 and a diagnostic frequency of 1 000. Resulting posterior probabilities were inspected for convergence in the program Tracer v1.6 [59] for MacIntosh.

### Ancestral character state reconstruction

Ancestral characters state reconstructions were performed on the resulting strict consensus tree from the parsimony analysis as well as on the All Compatible Clades consensus tree from the Bayesian analysis. For the strict consensus tree, ancestral character state reconstruction analysis was performed on the braincase characters (characters 93, 109, 110, and 111) using Mesquite version 3.10 build 765 [60] and were estimated using both parsimony and the maximum likelihood (Mk1) statistical model, which calculates the proportional likelihoods of each character state at ambiguous nodes. For the All Compatible Clades tree generated by the Bayesian analysis, the evolutionary scenarios for the braincase characters were again analysed with maximum likelihood ancestral reconstructions, with the assumption of equal rates of evolution. This method was completed in R [61] using ACE (ancestral character estimation) and phytools packages [62]. This dual approach for ancestral character state reconstruction follows the currently widely accepted methodologies in the literature [21, 63–67].

## Results

### Phylogenetic analysis

**Fig 2:**
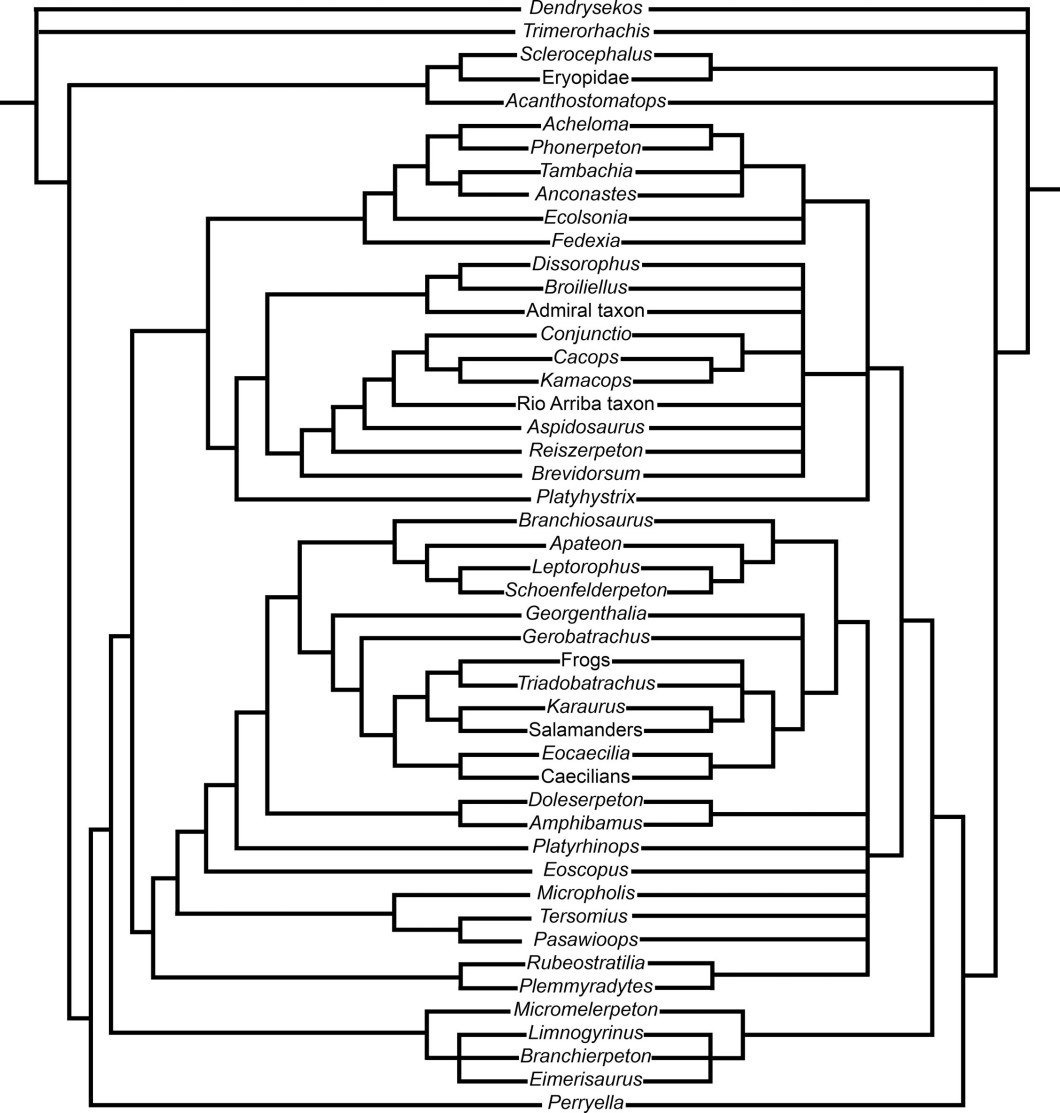
The results of the phylogenetic analyses of Dissorophoidea. The Bayesian analysis All Compatible Clades tree (left) obtained from MrBayes and the strict consensus tree (right) of 81 922 most parsimonious trees obtained from PAUP.

The analysis ran in PAUP resulted in 81 922 most parsimonious trees, each with 345 steps in length (consistency index, 0.3623; retention index, 0.7287; Fig 2, right). The strict consensus tree from the PAUP analysis is overall poorly resolved at the finer taxonomic levels (Fig 2, right), whereas the All Compatible Clades consensus tree from the Bayesian analysis has virtually no polytomies (Fig 2, left). The broader family level divisions, and subgroupings within, are very similar between both analyses (Fig 2). For example, in both analyses, the Amphibamiformes form a clade that includes Amphibamidae, Branchiosauridae, and a monophyletic Lissamphibia. In both analyses caecilians and *Eocaecilia* are found to be the sister group of Batrachia; however, in the parsimony analysis *Karaurus* plus salamanders form an internal clade that occurs in a polytomy with frogs and *Triadobatrachus*. *Gerobatrachus* forms the sister taxon to Lissamphibia in the Bayesian analysis (Fig 2, left), whereas in the parsimony analysis *Gerobatrachus*, *Georgenthalia*, and Lissamphibia are recovered in a polytomy (Fig 2, right). The Dissorophidae and Trematopidae clades that make up the Olsoniformes are recovered here, although the former is poorly resolved internally in the parsimony analysis. In both analyses the Olsoniformes is the sister clade to the Amphibamiformes (Fig 2, right). Also, in both analyses, Micromelerpetidae is found to be the sister group to the Olsoniformes and Amphibamiformes clade and represents the basalmost branch within Dissorophoidea. *Perryella* is the sister taxon to Dissorophoidea. One node below this, *Sclerocephalus* and Eryopidae form a clade in both analyses, with *Acanthostomatops* on their stem. Finally, in both analyses, *Trimerorhachis* forms a polytomy with the outgroup *Dendrysekos*, at the base of the tree.

### Ancestral character state reconstruction

Ancestral character state reconstructions were conducted for the braincase characters only (characters 93, 109, 110 and 111) and were estimated using both parsimony and maximum likelihood on the results of the parsimony analysis, and maximum likelihood on the results of the Bayesian analysis. The results of all three methodologies revealed the lineage leading to crown Lissamphibia is characterized by three absences that are identified to be secondary losses within the Amphibamiformes clade. These losses were found at the same nodes using the maximum likelihood methods for the parsimony and Bayesian trees; however, some of these losses were ambiguous using parsimony methods of ancestral state reconstruction. We summarize the results for all three methods below.

**Fig 3:**
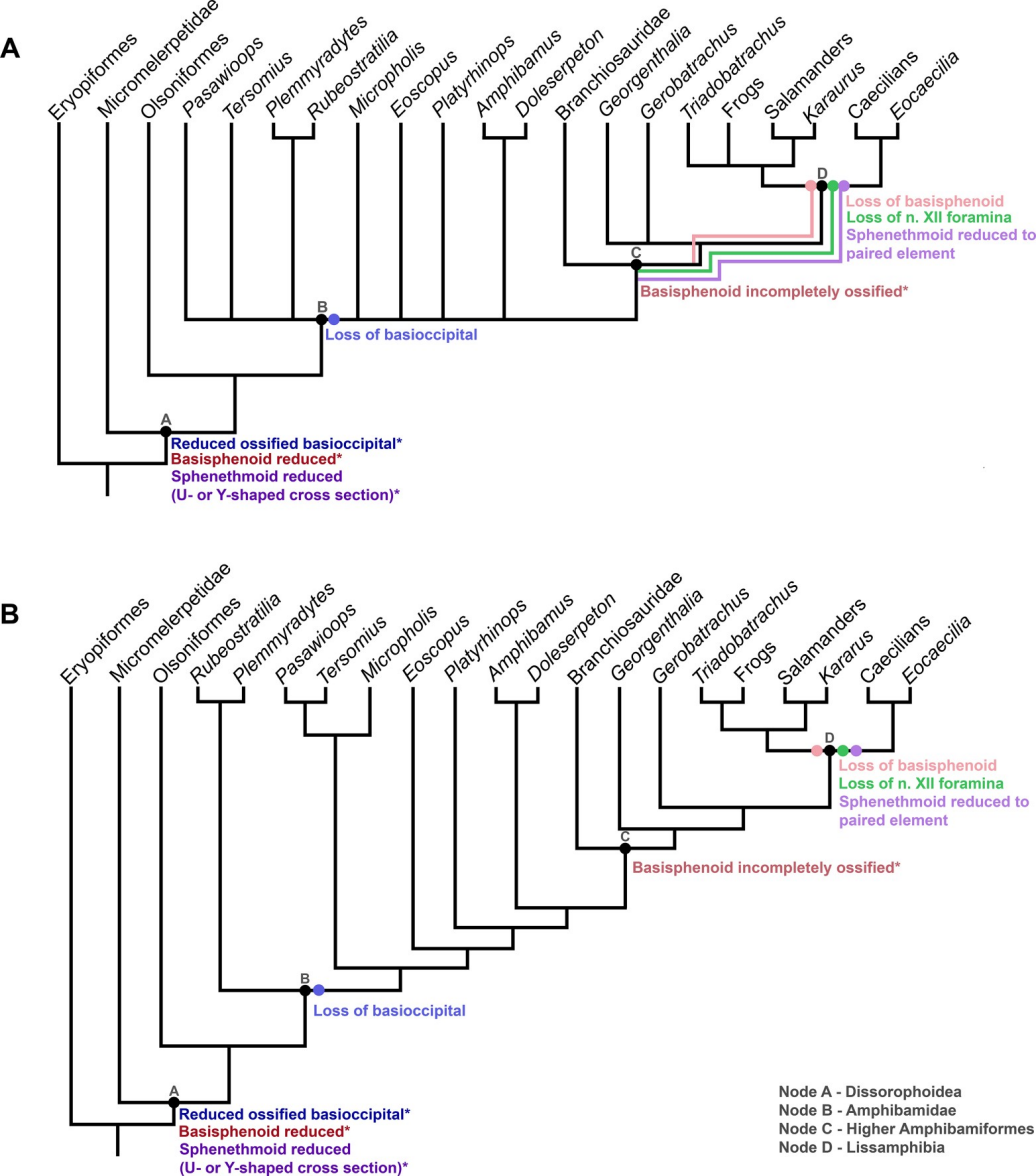
Summary of the ancestral character state reconstructions of the braincase characters on slightly pruned trees derived from both parsimony and Bayesian analyses. A) Parsimony and maximum likelihood ancestral character state reconstructions of the braincase characters on the strict consensus tree derived from the parsimony analysis. Coloured lines represent zones retrieved as ambiguous for the optimization of each character state under the parsimony criterion, and dots represent the locations retrieved as most probable location for each character state. B) Maximum Likelihood ancestral character state reconstructions of the braincase characters on the All Compatible clades consensus tree derived from the Bayesian analysis. The discrete states of the four characters used in this analysis (93, basioccipital loss in blue; 109, basisphenoid loss in red; 110 hypoglossal nerve, n. XII, foramina lost in green; 111, sphenethmoid reduced to paired structures in purple) are described in the locations where states were optimized. Other reductions that are described in the text, but unable to be confirmed in fossil taxa (certain potential reductions) are indicated with asterisks.

The first is the complete loss of the basioccipital (i.e. of both the ossified element and the cartilaginous precursor), which occurs at the base Amphibamiformes (Fig 3, Node B). The basioccipital is retained in all other lineages studied here. The loss of the basioccipital at the base of Amphibamiformes (Fig 3, Node B) was reconstructed as unambiguous using parsimony and with a proportional likelihood of 0.99 using maximum likelihood on the strict consensus tree from the parsimony analysis. This is the same location reconstructed by maximum likelihood on the Bayesian tree with a likelihood value of 0.41 at the base of Amphibamiformes (Fig 3, Node B), and with a likelihood value of 0.92 at the node that includes all Amphibamiformes except *Rubeostratilia* and *Plemmyradytes*.

The second loss occurs in the higher Amphibamiformes, where an ossified basisphenoid is lost (Fig 3, Node C). Branchiosaurids apparently have a cartilaginous basisphenoid [48–49], and in lissamphibians the basisphenoid is absent as either a cartilaginous or ossified element (Fig 3, Node D). The parsimony reconstruction failed to resolve an unambiguous location of the complete loss of the basisphenoid on the strict consensus tree from the parsimony analysis (Fig 3A). As a result, the zone of ambiguity spans the nodes that include the branching of *Georgenthalia*, *Gerobatrachus* and Lissamphibia. The maximum likelihood reconstruction on the strict consensus tree recovered the complete loss of the basisphenoid as occurring at the base of Lissamphibia (Fig 3, Node D) with a proportional likelihood of 0.97. This is the same location obtained for the maximum likelihood reconstruction on the Bayesian-derived tree with a likelihood value of 0.54.

The parsimony reconstruction again failed to resolve an unambiguous location for the location of the loss of foramina for cranial nerve XII and the reduction of the sphenethmoid to small, paired elements (Fig 3). The zone of ambiguity for both of these characters spans the nodes that include the higher amphibamids (Branchiosauridae, *Georgenthalia*, and *Gerobatrachus*; Fig 3A, Node C). The maximum likelihood reconstruction on the strict consensus tree recovered the loss of cranial nerve XII foramina and the sphenethmoid change at the base of Lissamphibia (Fig 3, Node D) with a proportional likelihood of 0.93 for the former and 0.96 for the latter. This is the same location for both of these character state changes obtained for the maximum likelihood reconstruction on the Bayesian-derived tree with a likelihood value of 0.61 for the loss of cranial nerve XII foramina and 0.81 for the change to the sphenethmoid.

## Discussion

Here we produce a revised, broadly sampled matrix that includes phylogenetically informative braincase characters for the inference of the intrarelationships of Dissorophoidea, and for the investigation of braincase evolution in the lineage leading to modern amphibians. Our extensive matrix samples from all dissorophoid clades and is inclusive of both Olsoniformes and the Amphibamiformes lineages of Dissorophoidea. Additionally, representative members from earlier diverging temnospondyl lineages have been included here for completeness and context. Although the Bayesian Inference tree is well resolved, the parsimony tree reveals some problems may still exist with regards to resolving the relationships within the smaller clades. With overall patterns of relationships largely congruent between analytical methods, it is hoped that this matrix, with its wide sampling of taxa and characters, provides a baseline for future analyses of this important group. As this analysis builds on other, previous phylogenetic analyses for lissamphibian origins, only a few key aspects of the resulting topology are noted here.

Overall, the addition of the braincase characters did not cause the topology of the phylogeny to change much relative to topologies found in other morphological analyses, such as those completed by Schoch [7], which was the basis for this analysis, Maddin *et al*. [31], Schoch [56], Fröbisch and Schoch [19], Fröbisch and Reisz [68], and Huttenlocker *et al*. [69]. One difference in topology was the recovery of *Gerobatrachus* as the closest relative to Lissamphibia in our analysis, as opposed to its position as a stem batrachian recovered by Maddin *et al*. [21] and Anderson *et al*. [11]. However, this position is identical to that found by Schoch [7], where *Gerobatrachus* and *Georgenthalia* form a polytomy with Lissamphibia. Classically, *Doleserpeton* has been found as the most closely related amphibamid to Lissamphibia [11,21]. In the current analysis, *Gerobatrachus*, *Georgenthalia*, and Branchiosauridae are all found as more closely related to Lissamphibia than *Doleserpeton*. Interestingly, the present analysis obtains Branchiosauridae nested within the group traditionally referred to as Amphibamidae (Fig 2). This relationship differs from the sister taxon relationship between Amphibamidae and Branchiosauridae, which has previously been found in other analyses [11,16,19,21,33,70]. However, see Schoch [7] for a revised, more exclusive, definition of Amphibamidae that maintains its monophyly in this topological arrangement. Together the Amphibamidae, Branchiosauridae and previously recognized amphibamids form the group recently named Amphibamiformes [7].

For the olsoniform portion of the tree, the traditional division of Olsoniformes into trematopids and dissorophids is found (Fig 2). Unlike in the Maddin *et al*. [31] and Schoch [56] analyses, where *Platyhystrix* is found as the sister taxon to the armored dissorophids, here *Platyhystrix* has a much more basal position as the sister taxon to the other Olsoniformes in the parsimony analysis. Interestingly, earlier analyses of Dissorophidae were better resolved than the present parsimony analysis. This suggests the inclusion of additional olsoniform and amphibamiform taxa is causing topology conflicts as adding only braincase characters to Schoch’s [7] matrix did not change the topology (tree not shown). Detailed work on braincase anatomy in these taxa, and the many poorly understood dissorophids that we chose to include, may help resolve these polytomies in future studies using parsimony. These issues, however, were not found in the Bayesian analysis, suggesting future study into various factors influencing the systematic approaches may also lead to improved resolution in a parsimony analysis.

In terms of braincase evolution, a previous, qualitative analysis of braincase transformations in the lissamphibian lineage led to the hypothesis that a series of secondary losses characterized the origin of the form of the lissamphibian braincase [42]. This evolutionary pattern, in addition to the absence of a supraoccipital generally accepted for all temnospondyls at the time, was suggested to be consistent with an amphibamid temnospondyl origin of Lissamphibia [42]. However, the braincase loss characters were never actually added to a matrix, and thus, their distribution had not been rigorously tested in an analysis of all of the characters. The quantitative analyses performed here has, for the first time, demonstrated and incorporated into evolutionary hypotheses that the lissamphibian braincase is the product of a stepwise sequence of reductions and losses, confirming it is not the ancestral condition for tetrapods and is indeed secondarily derived in its reduced state [37,42,54,71].

We propose the following stepwise sequence of loss events in the origin of the form of the lissamphibian braincase. In several cases, additional reductions in the size and extent of ossifications also occur within each loss or reduction event. Some of these would be impossible to determine with certainty in the fossil record, and so were not included in the character definitions, but are instead mentioned here. The first is the reduction of the basioccipital from an ossified element to an absent element at the base of the amphibamiform clade (Fig 3, Node B). Temnospondyls have generally been noted to have a reduced basioccipital that fails to contribute to the occipital condyle in many lineages, however the degree of ossification is variable. For example, *Edops*, a basal temnospondyl, has a large, heavily ossified basioccipital that participates in the occipital condyle [43–44]. Schoch [41] noted that the basioccipital is reduced from a large element in taxa such as *Eryops*, to a smaller element in dissorophoids (Fig 2, Node A), where the basioccipital makes minimal contributions to the occipital condyles and is reduced in the posterior braincase region. Both *Micropholis* [34] and *Pasawioops* [68] (basal Amphibamiformes) have been described as possessing a cartilaginous basioccipital, and *Doleserpeton* [20] is described as having a basioccipital that is impossible to distinguish from the exoccipitals. In *Doleserpeton* this is perhaps indicative of the complete absence of the basioccipital bone and cartilage. Schoch [7] coded *Tersomius*, *Micropholis*, *Eoscopus*, *Amphibamus*, and *Doleserpeton* as lacking a basioccipital, noting this includes loss of the basioccipital cartilage. Additionally, adult branchiosaurids [7] lack a basioccipital bone. However, the character state may be more variable for extant lissamphibians because although many sources note the absence of an ossified basioccipital, and its cartilaginous precursor [6,7,40], some older descriptions indicate a cartilage referred to as a basioccipital in some frogs and less often in salamanders ([72] and sources synthetized within [73]). Most recent descriptions do not mention a basioccipital as contributing to the chrondrocranium or the ossified braincase in lissamphibians (e.g. [74–77]). Clearly this region of the skull requires further investigation to determine the homology of the structure termed basioccipital by some authors and determine with certainty if this element is completely lost (including cartilage precursor) in extant lissamphibians. In the meantime, based on more recent assessments of its presence/absence, the ancestral character state reconstructions unanimously placed the complete loss of the basioccipital at the base of Amphibamiformes.

Next is a reduction of the basisphenoid. The basisphenoid typically forms the osseous walls and support to the hypophyseal region of the brain (i.e., sella turcica) [37,78]. The basisphenoid also undergoes a reduction from an extensive element in *Eryops* to a smaller element in the dissorophoid *Kamacops* (Fig 3, Node A) and then to a rudimentary ossified element in the amphibamid *Tersomius* (Fig 3, Node B) [41]. While branchiosaurids have a cartilaginous basisphenoid [48–49], lissamphibians have been described as lacking the basisphenoid, including its cartilaginous precursor [40,50]. Data concerning the basisphenoid are unfortunately not available for other higher Amphibamiformes, *Georgenthalia* and *Gerobatrachus*. Our analysis suggests this loss took place at the base of Lissamphibia (Fig 3, Node D). However, the reduction of the basisphenoid may have been more gradual before its eventual loss. This is difficult to characterize in the amphibamid lineage, as it is an internal structure often not visible in many taxa but may improve as more CT datasets become available.

The loss of foramina for the hypoglossal nerve (n. XII) on the exoccipitals also occurs at the base of the clade containing Lissamphibia (Fig 3, Node D). This final loss is consistent with the trend of braincase reduction, as it implies a reduction in the number of occipital somites that would have enclosed n. XII within the braincase, as in amniotes [42,79]. All dissorophoids (except for lissamphibians), and all tetrapods in general, possess foramina for n. XII. Developmentally this is interpreted as corresponding to the presence of an amniote-like number of occipital somites contributing to the braincase (e.g., four or more) [37,51,79]. In lissamphibians, n. XII occurs posterior to the braincase due to the lack of incorporation of the somites associated with it (e.g., anteriormost three or fewer) [37,51,80–81]. This appears to be a trait unique to lissamphibians in this analysis, but arguably is not determinable in *Gerobatrachus*.

The final aspect of braincase reduction investigated in the present analysis is the transformation of the sphenethmoid from a large, single median U or Y-shaped element to a paired, smaller element in lissamphibians (Fig 1). In basal temnospondyls (e.g. *Edops*, *Eryops*), the sphenethmoid is a broad element [43–44]. In *Eryops* the sphenethmoid is a large, robust element that is fused with the basisphenoid and, together with the lateral sphenoids, it covers the entire midbrain and anterior brain regions, enclosing cranial nerve II and possibly other nerves [82]. The dissorophoid sphenethmoid is less robust, less well-ossified posteriorly and open dorsally yielding a U-shape (e.g. *Doleserpeton*, *Acheloma*) or Y-shape (e.g. *Rubeostratilia*) in cross-sectional outline (Fig 3, Node A) [20,35,83]. The sphenethmoid of all members of Lissamphibia is reduced to a pair of small bones that lack a ventral floor adjoining left and right sides. Even in caecilians and *Eocaecilia*, where what is termed the sphenethmoid has a median component in the anterior region (e.g. the unpaired mesethmoid and basiethmoid), the posterior portion that is homologous with the sphenethmoid of other taxa (i.e. the orbitosphenoid portion) is a paired element that lacks an ossified ventral floor [21,50,84–85]. In frogs and salamanders, the element is so reduced that only the small elements in the interorbital region remains [85]. The analysis performed here reveals the absence of an ossified ventral floor is a loss that occurs at the base of Lissamphibia (Fig 3, Node D). It further appears as though the cartilaginous precursor of the ventral portion of the sphenethmoid is also lost in lissamphibians [37,86]. This latter feature would be difficult to evaluate in fossil taxa, and so the distinction between sphenethmoid ventral cartilage present but not ossified, and cartilage totally absent could not be made here in the character definitions.

The reduction of all of these elements in temnospondyls and in dissorophoids has been used as support of a temnospondyl ancestry for lissamphibians, as lepospondyls do not show the same reductions in the braincase during their evolution [6,21,42]. Many lepospondyls do share a number of losses with lissamphibians, however, these tend to be components of the dermal skull [87], which experience higher rates of homoplasy than the braincase. Of further importance is the result that each of the loss events identified here currently appear to be unique to the lissamphibian lineage and have occurred in the braincase, a region known to have a stronger phylogenetic signal than other regions of the skull or postcranium [21,42,88–90]. We admit that the loss of non-braincase skull bones is something that happens commonly over the course of tetrapod evolution. For example, in addition to the losses discussed here, all lissamphibians or members within Lissamphibia have lost numerous skull and lower jaw bones additional to those described in the current analysis, including the jugals, supratemporals, intertemporals, postfrontals, postorbitals, postparietals, tabulars, coronoids, splenials, and surangulars, among others [22,49,91]. These widespread losses often resemble losses in other tetrapod lineages, but have happened in a convergent context. This context is especially relevant when similar processes, such as certain heterochronic processes or body size reduction, also happen convergently and bring along with them their correlated morphological outcomes. This is particularly important here because 1) the origin of the lissamphibian form has been tied to heterochronic processes that bear predictable outcomes (i.e., paedomorphosis) and 2) controversy surrounding their origins has been complicated by a lack of knowledge regarding which of these predictable features are homologous versus homoplastic.

For lissamphibians, heterochronic processes leading to paedomorphosis have long been suggested to have been important drivers in the origin of their form [1,3,23,49]. In a careful analysis, Schoch [92] demonstrated the extent to which similar morphologies could be produced when widely separated lineages undergo similar heterochronic processes, and additionally when these lineages undergo miniaturization. That the loss events noted here in the lissamphibian braincase do not seem to be homoplastic with most other Paleozoic tetrapods that also underwent similar heterochronic processes such as miniaturization [19,92] speaks to the resilience of the braincase against such developmental perturbations and to their promising indications of an amphibamiform affinity.

The patterns of reduction and ultimately morphological and compositional simplification noted here as characterizing the origin of the lissamphibian braincase is not limited to this part of the body. There are numerous examples of additional reduction and losses of the dermal elements contributing to the skull as well as the dentition leading to lissamphibians [22,49,91]. For example, the loss of dentition on the basal plate of the parasphenoid, and the fangs on the palatal elements (e.g. vomer, palatine, and ectopterygoid) are all lost at various stages within Amphibamidae leading to lissamphibians. The driving forces behind the unique pattern of braincase reduction in lissamphibians, in spite of the resilience of this region to morphological change, are ultimately unknown and our research represents an important first step in understanding the unique lissamphibian body plan. Importantly, our analysis contributes a piece to the broader story of simplification in the origin of lissamphibian form in general.

## Conclusions

The analysis performed herein demonstrated that the reduced condition of the lissamphibian braincase is a derived state compared to its closest temnospondyl relatives. Our broadly sampled phylogenetic analysis reveals the stepwise sequence of three loss events and one reduction as part of the evolutionary transformation leading to the origin of the form of the lissamphibian braincase. Furthermore, the significant steps in the sequence, as well as the sequence of braincase reduction itself appears to be unique to lissamphibians, consistent with an amphibamid temnospondyl origin. While previous research in early tetrapods (e.g. stereospondyls) has indicated that the degree of braincase ossification has little phylogenetic significance [45,71], in more recent years and in many lineages (e.g. lissamphibians, acanthodians, and mammals), the braincase has revealed itself to be an important tool for phylogeneticists, as it is strongly influenced by early developmental constraints and appears to be less influenced by external factors than other skull components (e.g. the craniofacial skeleton) [21,42,88–90]. Our research shows that the braincase is indeed a useful tool for at least the temnospondyl lineage leading to lissamphibians. The future addition of more braincase characters, especially those that may be more phylogenetically informative, will permit an even more detailed analysis of this transformation, and will help resolve details of the phylogenetic relationships within this important clade of tetrapods.

## Supporting information

S1 Appendix A list of characters used in the phylogenetic analyses. (DOCX)

S2 Appendix A list of taxa used in the phylogenetic analyses. (DOCX)

S1 Dataset The matrix used in the phylogenetic analyses. (NEX)
