# Supplementary material for: Braincase simplification and the origin of lissamphibians
Source: PLoS One. 2019 Mar 22;14(3):e0213694. doi: 10.1371/journal.pone.0213694 (PMC6430379; doi:10.1371/journal.pone.0213694)
Supplement: S1 Appendix — (DOCX) [file pone.0213694.s001.docx]

**S1 Appendix – Character List**

**1. Laterally exposed palatine** (LEP). Palatine overplated by jugal and lacrimal with no lateral exposure (0); palatine wedging between lacrimal and jugal to make contribution to skull roof and orbital margin (1); maxilla contributes to orbital margin by separating jugal and lacrimal in the absence of lateral exposure of palatine (2) (based on Sumida et al., 1998; Schoch and Rubidge, 2005); lateral exposure of palatine present and excluded from orbital margin by jugal and lacrimal contact (3). An LEP was shown by Boy (1995) to be present in adults of *Micromelerpeton credneri*, and it is also likely to exist in adult branchiosaurids (*Apateon gracilis*), but coded “?” because of disarticulation.

**2. Dorsal quadrate process** (DQP). Quadrate having smooth posterodorsal side (0); quadrate with prominent dorsoposterior outgrowth, the quadrate process (1). With the exception of larval-neotenic taxa (Branchiosauridae, Micromelerpetidae), the derived condition has been reported from all dissorophoids (Bolt, 1969; Schoch and Rubidge, 2005).

**3. Vomerine depression.** Ventral surface of vomers flat and element divided into anterior and posterior portion by transverse ridges that may or may not bear transverse tooth row (0); single unpaired depression in anterior portion of vomers that may or may not house an opening (1). (Schoch and Rubidge, 2005).

**4. Parasphenoid dentition.** Basal plate of parasphenoid bearing shagreen of small teeth (denticles) anteromedially (0); plate entirely smooth (1) (Schoch and Rubidge, 2005). In the Dissorophoidea, the derived condition is reported from two different families, the Branchiosauridae (Boy, 1972, 1987) and Dissorophidae (Berman et al., 1985).

**5. Parasphenoid denticle field.** Well established, with triangular outline and with apex reaching onto base of cultriform process (0); denticle field greatly expanded anteriorly to cover most of the cultriform process (1) (Schoch and Rubidge, 2005). Within Dissorophoidea, the derived condition is only known in some amphibamids (*Micropholis*, *Pasawioops*, *Amphibamus*, *Platyrhinops*). and *Eocaecilia*.

**6. Parasphenoid basal plate.** Roughly quadrangular dimensions, as long as wide (0); basal plate much shorter than wide, reaching about half the width (1). (Schoch and Rubidge, 2005).

**7. Vomerine denticle field.** Vomer covered with a more or less dense shagreen of teeth in addition to obligatory fang pair (0); shagreen confined to juvenile stages and/or absent throughout ontogeny (1). (Schoch and Rubidge, 2005).

**8. Vomerine fangs.** Vomer lacking fangs in its medial portion, outside lateral tooth arcade, but having smaller accessory teeth in that region (0); vomer with additional fang pairs posterior to midvomerine depression (1). (Schoch and Rubidge, 2005).

**9. Pterygoid-vomer contact.** Suture between pterygoid (palatine ramus) and vomer (0); pterygoid contacting only palatine (or ectopterygoid) and lacking suture with vomer (1). (Schoch and Rubidge, 2005).

**10. Pterygoid flange.** Palatine ramus of pterygoid merging continuously into basipterygoid ramus (0); palatine ramus broadening abruptly to form a pronounced transverse flange, giving the lateral margin a rectangular shape (1). The newly-defined apomorphic state is only present in dissorophids; in trematopids and amphibamids, the flange is rounded. (Schoch and Rubidge, 2005).

**11. Palatine, ectopterygoid.** Palatine and ectopterygoid much wider than maxilla (0); palatine and ectopterygoid reduced to narrow struts not wider than adjoining maxilla (1). The two bones are here considered together, because their width evolved in parallel and was probably linked. (Schoch and Rubidge, 2005).

**12. Interpterygoid vacuity.** Roundish or oval in outline (0); greatly expanded laterally at mid-level (1). (Milner, 1993).

**13. Narial flange.** Ventral (inner) side of prefrontal, lacrimal, and nasal smooth (0); inner side of these bones forming complicated bar-like structure (narial flange), permitting contact with antorbital bar (1). (Boy, 1981).

**14. Prefrontal process.** Prefrontal forming simple suture with lacrimal laterally (0); prefrontal underplating lacrimal widely by means of ventral prefrontal process contacting palatine (1). (Boy, 1981; Werneburg, 1989).

**15. Tabular size.** Tabular narrower than postparietal, but reaching almost same size as latter (0); tabular minute and laterally constricted by unique enlargement of otic notch (1). (Schoch and Rubidge, 2005).

**16. Tabular-squamosal contact.** Tabular and squamosal widely separated by supratemporal (0); squamosal meeting tabular, excluding supratemporal from otic notch (1). (Yates and Warren, 2000; Schoch and Rubidge, 2005).

**17. Postparietal length.** Postparietal forming transversely rectangular or quadrangular element (0); postparietal abbreviated and reduced to narrow, poorly ornamented strut at posterior margin of skull table (1). (Schoch and Rubidge, 2005).

**18. Squamosal-supratemporal suture.** Nearly as long as supratemporal itself (0); or shorter, reaching only one third or less of length of supratemporal (1). (Schoch and Rubidge, 2005).

**19. Supratympanic flange** (=semilunar flange in Schoch and Rubidge, 2005, terminology following Bolt, 1974a). Squamosal continuously ornamented around margin of otic notch (0); squamosal having dorsally exposed and ornamented area (supratympanic flange) stepping abruptly into steeply aligned, poorly ornamented portion (1).

**20. Semilunar flange** (=supratemporal flange of Schoch and Rubidge, 2005). Supratemporal without ventral projection into otic notch (0); supratemporal forming marked ventral flange participating in medial bordering of otic notch (1).

**21. Prefrontal-postfrontal.** Firmly sutured on the dorsal side, excluding the frontal from the orbital margin (0); both elements separated by frontal, at least dorsally (1). Whereas this character varies in some capitosaurs (Schoch, 2000), it has not been found to do so in dissorophoids. Notably, the stratigraphically earliest micromelerpetontids (*Limnogyrinus elegans*), branchiosaurids (*Branchiosaurus salamandroides*) and amphibamids (*Amphibamus grandiceps*, *Platyrhinops lyelli*) have the plesiomorphic condition, whereas this is not known from any trematopid or dissorophid.

**22. Interorbital width.** Narrow to moderately wide interorbital region in the 0.2–0.24 range (interorbital width/skull length) (0); or substantially wider (0.27–0.33) (1). In the outgroups, *Dendrysekos* has an unusually wide interorbital region, which is why *Sclerocephalus* has been taken as guide to the primitive condition. (Modified from Anderson et al., 2008b).

**23. Palpebral ossifications.** No ossifications other than sclerotic ring (0); numerous palpebral ossicles at medial margin of sclerotic ring (1). This character, first documented by Credner (1881, as “*Skleralpflaster*”) in *Branchierpeton* and described in detail by Boy (1972) in *Micromelerpeton*, was later emphasized by Daly (1994) as typical of amphibamids. However, it is not confined to Micromelerpetidae and Amphibamidae, but also known from *Broiliellus brevis* (Schoch 2012) and probably widespread among dissorophoids (Holmes et al., 2013). Palpebral ossicles are not to be confused with dentigerous platelets which cover the interpterygoid vacuities of many temnospondyls, a character that was much more widespread and is not considered here.

**24. Stapes.** Stapes with pronounced dorsodistal curvature directed towards dorsally located otic notch (0); or abbreviated without dorsodistal curvature, directed mostly laterally towards vertically aligned otic notch (1). The derived character-state is present in branchiosaurids, amphibamids, trematopids, and dissorophids (Schoch, 2012; Schoch and Anderson, 2016).

**25. Prefrontal-jugal contact.** Absent (0); present (1). The derived state is found in *Sclerocephalus* (representing the vast stereospondylomorph clade and eryopids that all share this feature). The resulting pattern in which the lacrimal does not border the orbit evolved convergently in *Cacops morrisi* (Reisz et al., 2009), where it results from an anterior expansion of the LEP, unlike in *Sclerocephalus* where the jugal extends further anteriorly.

**26. Maxilla dentition.** Extending posterior to the level of the posterior margin of the orbit (0); terminating at the level of such margin or anterior to it (1). This is an exclusive dissorophid character, which best seen in *Cacops* (Schoch, 2012).

**27. Skull outline.** Absence (0) or presence (1) of inward inflection of skull outline in dorsal view at the level of the maxilla-premaxilla suture. (Schoch and Rubidge, 2005).

**28. Septomaxilla.** At posterior rim of naris (0); at mid-level of naris, pointing inside (1). The derived state is present in all trematopids and dissorophids, differing from all other temnospondyl groups. (Schoch, 2018).

**29. Parietal width.** More (0) or less (1) than two and a half times as long as wide. (Schoch and Rubidge, 2005).

**30. Postparietal length.** Postparietals less (0) or more than (1) four times wider than long. (Schoch and Rubidge, 2005).

**31. Postorbital.** Narrowing to an acute posterior point (0); not narrowing, ending blunt (1).

**32. Vomer (posterior projection).** Absent (0) or present (1). (Schoch, 2018).

**33. Vomer (tooth row).** Vomer with (0) or without (1) a toothed, raised crest running anteroposteriorly and lying mesial to the choana. (Schoch and Rubidge, 2005).

**34. Palatine and interpterygoid vacuity.** Palatine partially or fully excluded from vacuity by pterygoid (0); bordering vacuity along its entire medial margin (1). (Reformulated from Milner, 1990; Yates and Warren, 2000)

**35. Cultriform process.** Moderately wide and flat on ventral side (0); throughout thin and round in cross-section (1). (Schoch, 2012).

**36. Exoccipital-tabular contact.** Absent (0); present (1). The derived state is a synapomorphy of eryopiforms (*Sclerocephalus*) and also reported in zatracheids. (Boy, 1990).

**37. Exoccipital-postparietal contact.** Present (0); absent (1). (Coding reverted from Polley and Reisz, 2011), because absence is clearly exclusive to cacopines in the present set of taxa).

**38. Position of jaw articulation.** Posterior to (0), level with (1), or anterior to (2) the posterior facets of the exoccipitals. (Yates and Warren, 2000).

**39. External narial opening.** Uniform, oval shaped margin (0); posteriorly expanded at the expense of lacrimal, with distinct anterior and posterior regions giving external naris an overall ‘key-hole’ shape (1) (Dilkes, 1990).

**40. Internarial fenestra.** Absent (0); present (1) (Dilkes, 1990; Anderson et al., 2008b).

**41. Marginal teeth of upper jaw.** Uniform in size (0); caniniform teeth on premaxilla and maxilla (1) (Dilkes 1990).

**42. Prearticular.** Inflection of the prearticular along the medial rim of the adductor fossa: absent (0); present (1) (Dilkes, 1990).

**43. Ventral border of otic notch.** Slopes posteroventrally (0); nearly horizontal (1) (Dilkes and Reisz, 1987). Within the Trematopidae, this character may have been subject to ontogenetic change or scaling, as small trematopids have the plesiomorphic, large ones the apomorphic condition.

**44. Median vomerine septum.** Absent (0); present (1) (Dilkes and Reisz, 1987; Dilkes, 1990).

**45. Tabular process.** Any state between short and absent (0); curves gradually to meet robust quadrate process (1); bent down sharply at approximately a right angle to the dorsal edge of the skull table and fused to the quadrate process (2) (Dilkes and Reisz, 1987).

**46. Stapedial foramen.** Present (0); absent (1) (Boy, 1981; Daly, 1994). This character was long believed to separate trematopoids from dissorophids, with the latter considered to lack a foramen, However, *Cacops morrisi* clearly has a stapedial foramen (Gee and Reisz, 2018).

**47. Knobby exostoses (skull roof).** Absent (0); present (1) (Daly 1994). The derived state is found in dissorophids, some of which have excessive bone growth in the skull (DeMar, 1968).

**48. Subnarial lacrimal process.** Long (0); short (1) (Sumida et al., 1998).

**49. Squamosal.** Semilunar curvature of along ventral border of the supratympanic flange: absent (0); present (1) (Berman et al., 1985; Dilkes, 1990).

**50. Dorsal rim of occiput.** Gently ornamented (0); ornament forming marked transverse ridge (1). (Schoch, 2012).

**51. Ratio of preorbital length to postorbital length.** Preorbital length greater than postorbital length by more than 10% (0); preorbital and postorbital lengths approximately equal (1); postorbital length greater than preorbital length by greater than 10% (2). (Yates and Warren, 2000).

**52. Suborbital bar height.** Greater than 10% of the total midline skull length (0); less than 10% of the total midline skull length (1). (Yates and Warren, 2000).

**53. Minimum distance otic notch-posterior orbital margin.** Greater than 25% of the total midline skull length (0); between 10 and 25 % of the total midline skull length (1); less than 10% of the total midline skull length (2). (Schoch, 2012).

**54. Basipterygoid articulation.** Discrete facet for ball-and-socket joint or overlap (0); firmly sutured at mid-level of widened basipterygoid process (1). (Yates and Warren, 2000).

**55. Postorbital-supratemporal.** Sutured (0); separated by postfrontal (1). This character appears to be polymorphic in *Phonerpeton*, but obligate in *Cacops morrisi*; it is unknown in the heavily ornamented taxa (*Cacops aspidephorus*, *Kamacops*, *Zygosaurus*).

**56. Intertemporal.** Present (0); absent (1). Although a clear-cut character, the intertemporal was lost several times independently in basal temnospondyls. In *Sclerocephalus* and *Micromelerpeton*, it has been reported as a polymorphism in several samples (Boy, 1972, 1988).

**57. Osteoderms.** Median unpaired osteoderms (one per vertebra) absent (0); or present. (1). (Dilkes and Brown, 2007). DeMar (1966), who first studied the dissorophid armor in depth, distinguished two major types (see character 58), arguing for their independent evolutionary origin. However, there is no reason to conclude that the inner series of osteoderms is not homologous throughout dissorophids, just because its mode of articulation differs in sub-clades.

**58. Osteoderm width.** Narrow (0), wide (1). *Dissorophus* and *Broiliellus texensis* share wide (internal and external) osteoderms reaching at least the width of the two postparietals. In Aspidosaurus (sensu stricto) and *Cacops*, the osteoderms are much narrower, not exceeding the maximum width of the transverse processes (DeMar, 1966).

**59. Cranial ridges.** Dorsal surface of skull roof flush or with gentle ridges on prefrontal, orbital margin, and in the temporal region (0); or with pronounced ridges especially in the snout (1). Although *Cacops* and *Kamacops*, *Scapanops*, and *Conjunctio* also have these ridges, they are always shallower than in *Dissorophus* and the three *Broiliellus* species (Schoch, 2012).

**60. Ilium (dorsal process).** High and slender, distally not wider than shaft (0); or short and stout, not higher than base is wide, with broadened dorsal end (1). This is one of the more convincing characters shared by trematopids and dissorophids, as it never occurs in other dissorophoids (Daly, 1994; Schoch and Rubidge, 2005)

**61. Parasphenoid plate.** Lateral margin with or without moderate posterolateral projection (0); posterolateral wing expanded well beyond the level of the basipterygoid suture. Lateral projections of the basal plate are found in most amphibamids, branchiosaurids, and dissorophids, but they are most pronounced in *Broiliellus* and *Dissorophus*. The presence of a projection in state 0 is subject to ontogenetic changes and variation (Schoch, 2012).

**62. Carotid artery.** Exit foramina for carotid artery located on the parasphenoid plate (0); or at the base of cultriform process (1). The derived state is clearly present in Cacops morrisi and Kamacops, but unkown in other cacopines; it may thus have been more widespread within this group. *Dissorophus*, *Broiliellus*, *Aspidosaurus*, *Platyhystrix*, and *Conjunctio* show the plesiomorphic state (Shishkin, 1968).

**63. Tabular horn: posterior extension.** Round or pointed but short (0); or elongated, forming main portion of tabular (1). Within dissorophoids, the derived state is confined to the three *Broiliellus* species (Schoch, 2012).

**64. Jugal.** Dorsal surface of jugal regularly ornamented (0); or with large eminence bearing knobby ornament (1). This feature is most pronounced in *Dissorophus*, but also present in *Broiliellus brevis* (Schoch, 2012). It is unknown whether the derived state was age- or size-related, but it is certainly absent in *Cacops* and *Kamacops*.

**65. Parasphenoid: muscle scars.** The posterior and posterolateral parts of the parasphenoid plate bear gentle depressions or other types of muscle attachments (0); or they house deep pockets for such attachments (1). The derived state is present in *Dissorophus* and *Broiliellus* among the dissorophoids, but has also a wide distribution in stereospondyls (Watson, 1962; Yates and Warren, 2000).

**66. Pointed snout.** The tip of the snout is of various width but parabolic to square-shaped (0); or it is pointed (1). In dissorophoids, the pointed snout is shared by *Dissorophus* and *Broiliellus brevis* (Schoch, 2012).

**67. Interclavicle.** (0) Interclavicle rhomboidal, at least two times longer than wide. (1) Interclavicle with abbreviated anterior and posterior ends and as wide as long. (2) Interclavicle much shorter than wide, without anterior process. State 2 is probably derived from state 1, and present throughout branchiosaurids. Witzmann and Voigt (2015) have confirmed this coding in a recent review.

**68. Supinator process.** Anterodistal region of humerus with supinator process (0); or lacking such process (1). As emphasized by Milner (1990), trematopids share a supinator with more primitive temnospondyls, whereas dissorophids, amphibamids, and branchiosaurids lack it. Adult specimens of *Micromelerpeton* also have a supinator (Boy, 1995).

**69. Entepicondylar foramen.** Distal end of humerus pierced by an entepicondylar foramen (0); or lacking such opening (1). This is a consistent post-*Dendrysekos* feature (Milner, 1990).

**70. Dorsal eminences.** Skull table without major raised areas other than ornamenting ridges (0); or with elevated eminences on the frontal, parietal, postfrontal, and postparietal (1). The apomorphic state is confined to the three species of *Broiliellus*. (Schoch, 2012).

**71. Anterior trunk ribs.** The anterior trunk ribs have widened ends (0), or are simple rods without distal expansion (1). The apomorphic character-state is present in the amphibamids *Platyrhinops*, *Amphibamus*, and *Eoscopus*. (Schoch and Milner, 2008).

**72.** **Ventral bony scales.** Spindle-shaped ventral scales fully ossiﬁed (0), or absent (1). This is only recognized in large specimens, where osteoderms are fully formed (Boy 1987; Witzmann 2007). The plesiomorphic character state is present only in *Branchiosaurus*, while all other branchiosaurids lack spindle-shaped ventral osteoderms in adult stages. (Schoch and Milner, 2004, 2008).

**73. Branchial dentition.** Primitively, there are four rows of branchial denticles which are attached to rectangular or oval platelets bearing a total of 3–10 denticles that form a ratchet, as two of the outgroups indicate (0), or there are six rows of isolated denticles with a single point, or multi-ended (1). (Boy, 1972; Schoch and Milner, 2008). The branchial ossicles of amphibamids are still unknown, even in the larval specimens referred to *Amphibamus grandiceps* by Milner (1982).

**74.** **Basipterygoid joint.** Ossified (0), or unossified (1). The derived state is found in larval-neotenic micromelerpetids and branchiosaurids, but also occurs in adult stereospondylomorphs (Boy, 1988; Witzmann and Schoch, 2006).

**75. Humerus: morphology.** The humerus length:waist ratio is 3:4 in *Dendrysekos*, *Balanerpeton*, *Sclerocephalus* (0); 4:6 in *Micromelerpeton* and the branchiosaurid *Schoenfelderpeton* (1), and 6:10 in amphibamids and most branchiosaurids (2). We admit that this character may be problematic when the profound ontogenetic changes in large temnospondyls are considered (e.g., Pawley and Warren, 2006), but for comparisons among small dissorophoids and especially branchiosaurids it does appear to be informative. (Schoch and Milner, 2008).

**76.** **Presacral count: reduction.** Primitively a total of 24 presacral vertebrae are present (0), while fewer than 24 presacrals is clearly derived (1). All branchiosaurid species have fewer than 24 presacrals as an average, and most have only around 20 (Boy, 1972, 1978, 1987). The reduction of the presacral count to fewer than 24 is observed in the Amphibamidae as well, but there it is clearly a derived character-state characterizing *Amphibamus grandiceps* (Milner, 1982) and *Micropholis stowi* (Schoch and Rubidge, 1995), while the primitive condition for the family is 24 presacrals (Daly, 1994; Clack and Milner, 2010). A further derived state is the reduction to 17 presacrals or fewer, which is shared by *Gerobatrachus* and lissamphibians (2).

**77.** **Presacral count: elevation.** Twenty-four presacral vertebrae (0), or clearly higher count of 26–29 (1). This condition is obviously a separate evolutionary pattern, confined to the Micromelerpetidae, which is why it is here defined as a separate character. (Schoch and Rubidge, 2005).

**78. Bicuspidity.** The crowns of marginal and palatal teeth are primitively monocuspid (0), but are clearly bicuspid, with the cusps oriented lingually-labially (1), the derived state shared by some derived amphibamids and lissamphibians (Bolt, 1977).

**79. Pedicely.** The crowns of marginal and palatal teeth are primitively conical and continuous with the base (0), but are separated from the base by a zone in derived amphibamids, which is called pedicely (1) (Parsons and Williams, 1962; Bolt, 1969, 1977, 1979).

**80.** **Width of palatine and ectopterygoid.** These elements are either attached to the maxilla throughout their lateral margins (0), or the ectopterygoid and the posterior portion of the palatine are well separated from the maxilla, with the palatine attaining a Y-shaped outline (1). This character forms the second robust support for the monophyly of the Branchiosauridae. In addition to its uniqueness among temnospondyls, this state differs essentially from the derived condition in amphibamids where the palatine and ectopterygoid are extremely slender but attached to the maxilla, giving distinctly wider interpterygoid vacuities than in branchiosaurids or dissorophids, for instance. The peculiar branchiosaurid condition is acquired early in development (Schoch, 1992) and was maintained well into the largest growth stages (Boy, 1978; Werneburg, 1991).

**81.** **Ectopterygoid.** This forms a slender elongate element which bears teeth in the primitive condition, retained in branchiosaurids (0). In *Schoenfelderpeton* and *Doleserpeton*, it is reduced to a thin edentulous strut (1). (modified from Schoch and Rubidge, 2005).

**82. Width of maxillary shelf.** The main dentigerous body of the maxilla is slender in the primitive condition (0), while in *Apateon* it is markedly broadened, especially in adult and/or large specimens. (Schoch and Milner, 2008).

**83.** **Postorbital separated from supratemporal.** In the primitive condition, the supratemporal has a broad suture with the postorbital (0). In *Leptorophus* and *Schoenfelderpeton*, a posterolateral projection of the postfrontal keeps the tiny postorbital apart from the supratemporal (1), see Boy (1986) and Werneburg (2001).

**84. Pubis.** Ossified (0), or unossified (1). (Schoch, 2018).

**85.** **Intercentra.** In the primitive condition of temnospondyls, intercentra are wedge-shaped (0); in trematopids, they are dorsally enlarged (1). (Schoch, 2012).

**86.** **Pleurocentra.** Confined to the dorsal portion of the vertebral centrum (0), reaching ventrally down the flanks (1), forming short closed rings (2), forming elongate cylinders (3) (modified from Schoch and Rubidge, 2005).

**87.** **Otic notch.** Present (0), absent (1). An enlarged semilunar squamosal embayment is present in all early and basal temnospondyls, as well as the vast majority of dissorophoids. The derived state is established in most dvinosaurians (incipiently present in *Trimerorhachis*) (Schoch and Milner, 2014).

**88.** **Trunk ribs.** Moderately long and curved (<length of three vertebrae) (0), or short and straight (<3 vertebrae). (Schoch and Rubidge, 2005).

**89. Choana.** (0) Choana narrow, forming elongated oval with parallel, parasagittal lateral and medial margins. (1) Choana expanded anteromedially (Schoch, 1998; Schoch and Rubidge, 2005). The derived state is not present in branchiosaurids and some amphibamids, but in *Micropholis*, *Amphibamus*, *Doleserpeton*, *Gerobatrachus*, and lissamphibians.

**90.** **Cleithrum: head.** (0) Head of cleithrum aligned along anterior rim of scapula. (1) Cleithrum with posterodorsally enlarged head, wrapping around scapula dorsally. (Schoch and Rubidge, 2005).

**91. Cleithrum: size.** (0) Cleithrum with large dorsal head much wider than shaft. (1) Cleithrum forming simple rod, without any head. (Schoch and Rubidge, 2005).

**92. Scapula.** (0) Scapula forming low or moderately high element, depending on degree of ossification, about two times longer than wide. (1) Scapula dorsally much extended, being three to four times longer than wide. (Schoch and Rubidge, 2005).

**93. Basioccipital and supraoccipital.** Present (0), or absent (1). The derived state characterizes all lissamphibians and adult branchiosaurids (Schoch and Milner, 2004). In the extant taxa, neither bone nor cartilage is formed in these regions, indicating that it is not simply the failure of a cartilage to ossify, but the complete loss of the element. Instead, the basisphenoid, which is also absent is extant amphibians, is retained in branchiosaurids albeit incompletely ossified (Boy, 1972).

**94. Contact maxilla–quadratojugal.** These are either sutured in early ontogenetic stages (0), or growth of the maxilla is slowed down with late suturing (1), or elements fail to meet, leaving a gap in the cheek (2). The three states are obviously correlated with ontogenetic character transformation (2>1>0), and outgroup comparison indicates clearly that the adult state (0) is the primitive condition. (Boy, 1987).

**95.** **Medial suture of supratemporal.** The supratemporal is wide with a straight or convex medial suture (0), or much narrower and medially concave (1). The derived state is etablished in *Leptorophus* and *Schoenfelderpeton*. (Schoch and Milner, 2008).

**96. Shape of supratemporal.** Primitively, the anterior portion of the supratemporal is blunt and not much narrower than the greatest width of the element (0). In most *Melanerpeton*, *Leptorophus* and *Schoenfelderpeton*, the anterior end is pointed to give the element a triangular outline; the squamosal compensates this space with a medial projection (1). (Schoch and Milner, 2008).

**97. Jugal anteriorly abbreviated.** Irrespective of its lost contact to the lacrimal, the jugal may be either anteriorly long (0), or it may lack the anterior process and end bluntly (1).

**98. Intercentra.** Present (0), or absent (1). (Anderson et al., 2008a).

**99. Medial quadratojugal process.** Absent (0), present (1). (Boy, 1972).

**100. Pterygoid: slender.** Primitively, the pterygoid has wide tooth-bearing palatine and basipterygoid rami (0), in the derived condition, the pterygoid has extremely thin, bar-like rami (1). The derived condition is established in branchiosaurids, *Gerobatrachus*, *Triadobatrachus*, and *Karaurus*. (Schoch, 2018).

**101. Circumorbital bones: shape.** Plate-like (0), extremely thin, reduced to margin of orbit (1). Modified from Anderson et al. (2008b).

**102. Vomerine dentition.** Single row of larger teeth including tusk pair (0), or patch of tiny denticles (1). The derived state is found in *Doleserpeton*, *Amphibamus*, and *Gerobatrachus*, whereas branchiosaurids have the plesiomorphic state. This character is confined to the region of the posterolateral branch of the vomer, whereas character (7) concerns tooth patches on the main portion of the element, the anteromedial plate. (Bolt, 1979).

**103. Premaxilla: ascending process.** Plate-like, aligned posterodorsally (0), or thin and rod-like, oriented posteromedially (1). The derived state is present only in *Gerobatrachus* and batrachians. (Lebedkina, 1979).

**104. Preorbital region: width.** Of variable width, but with long axis of lacrimal aligned parasagittally (0), or much widened, and lacrimal aligned nearly transversely (1). (Schoch, 2018).

**105. Ectopterygoid.** Present (0), or absent (1). *Doleserpeton* retains an extremely thin, edentulous ectopterygoid (Sigurdsen and Bolt, 2010: Fig. 2B).

**106. Postfrontal, postorbital, jugal, supratemporal, postparietal, tabular.** Present (0), or absent (1). Schoch (2014d).

**107. Cleithrum.** Present (0), or absent (1). The derived state is confined to lissamphibians (batrachians in the present analysis). The presence of a cleithrum in anurans is highly controversial (Havelovká and Roček, 2006), and has therefore been coded as absent here.

**108. Interclavicle.** Present (0), or absent (1). The derived state is confined to lissamphibians (batrachians in the present analysis).

**109.** **Basisphenoid.** Present (0) or absent (1). (NEW)

**110.** **Exoccipital foramina for cranial nerve XII.** Present (0) or absent (1). (NEW)

**111.** **Sphenethmoid.** Sphenethmoid is either present as a single, medially positioned entity with the floor present (0), or the floor is absent and the sphenethmoid is present as two elements (1). (NEW)
