# Supplementary material for: Braincase simplification and the origin of lissamphibians
Source: PLoS One. 2019 Mar 22;14(3):e0213694. doi: 10.1371/journal.pone.0213694 (PMC6430379; doi:10.1371/journal.pone.0213694)
Supplement: S2 Appendix — (DOCX) [file pone.0213694.s002.docx]

**S2 Appendix - List of taxa and resources used in analysis**

| *Acanthostomatops vorax* [1] | *Acheloma cummiunsi* [2] | Admiral taxon [3] | *Amphibamus*  (YPM 794) |
| --- | --- | --- | --- |
| *Anconastes* [4] | *Apateon gacilis* [5] | *Ascaphus* [6]  (representative frog species) | *Aspidosaurus* [7] |
| *Branchierpeton amblystomum* [8] | *Branchiosaurus* [9] | *Brevidorsum* [3] | *Broiliellus* [3] |
| *Cacops* [10] | *Conjunctio* [3] | *Dendrepeton* [11,12] | *Dissorophus* [13] |
| *Doleserpeton* [14] | *Ecolsonia* [15] | *Eimerisaurus graumanni* [16] | *Eocaecilia* [6,17] |
| *Eoscopus* [18] | Eryopidae [19,20] | *Fedexia* [21] | *Georgenthalia* [22] |
| *Gerobatrachus* [23] | *Hynobius* [6] (representative salamander species) | *Kamacops* [24] | *Karaurus* [6,25] |
| *Leptorophus* [26,27] | *Limnogyrinus elegans* [28] | *Micromelerpeton* [29] | *Micropholis* [30] |
| *Pasawioops MCZ* (MCZ 1415, OMNH 73019, CT data) | *Perryella olsoni* [31] | *Phonerpeton* [32] | *Platyhystrix* [33] |
| *Platyrhinops* [34] | *Plemmyradytes* [35] | *Reiszerpeton*  (MCZ 1911) [36] | *Rhinatrema* (representative caecilian species) [6] |
| Rio Arriba taxon [3] | *Rubeostratilia* [37] | *Schoenfelderpeton* [26] | *Sclerocephalus* [38] |
| *Tambachia* [39] | *Tersomius* [40] | *Triadobatrachus* [6,41,42] | *Trimerorhachis insignis* [43] |

1. Witzmann F, Schoch RR. Skeletal development of the temnospondyl *Acanthostomatops vorax* from the Lower Permian Döhlen Basin of Saxony. Earth Environ Sci Trans R Soc Edinb. 2006;96: 365–185.
2. Polley BP, Reisz RR. A new Lower Permian trematopid (Temnospondyli: Dissorophoidea) from Richards Spur, Oklahoma. Zool J Linn Soc. 2011;161: 789–815.
3. Carroll RL. Early evolution of the dissorophid amphibians. Bull Mus Comp Zool. 1964;131: 161-250.
4. Berman DS, Reisz RR, Eberth DA. A new genus and species of trematopid amphibian from the Late Pennsylvanian of north-central New Mexico. J Vert Paleontol. 1987;7: 252–269.
5. Fröbisch NB, Schoch RR. The largest specimen of *Apateon* and the life history pathway of neoteny in the Paleozoic temnospondyl family Branchiosauridae. Foss Rec. 2009;12: 83–90.
6. Maddin HC, Jenkins FA Jr, Anderson JS. The braincase of *Eocaecilia micropodia* (Lissamphibia, Gymnophiona) and the origin of caecilians. PLOS One. 2012;7: e50743.
7. Berman DS, Lucas SG. *Aspidosaurus binasser* (Amphibia, Temnospondyli), a new species of Dissorophidae from the Lower Permian of Texas. Ann Carnegie. 2003;72: 241–262.
8. Schoch RR, Milner AR. Handbuch Der Paläoherpetologie: Encyclopedia of Paleoherpetology. Temnospondyli I/by Rainer R. Schoch & Andrew R. Milner. Pfeil; 2014.
9. Schoch RR, Milner AR. The intrarelationships and evolutionary history of the temnospondyl family Branchiosauridae. J Syst Palaeontol. 2008;6: 409-431.
10. Williston SW. *Cacops*, *Desmospondylus*; new genera of Permian vertebrates. Geol Soc Am Bull. 1910;21: 249–284.
11. Robinson J, Ahlberg PE, Koentges G. The braincase and middle ear region of *Dendrerpeton acadianum* (Tetrapoda: Temnospondyli). Zool J Linnean Soc. 2005;143: 577–597.
12. Holmes RB, Carroll RL, Reisz RR. The first articulated skeleton of *Dendrerpeton acadianum* (Temnospondyli, Dendrerpetontidae) from the Lower Pennsylvanian locality of Joggins, Nova Scotia, and a review of its relationships. J Vert Paleontol. 1998;18: 64–79.
13. De Mar RE. The Permian labyrinthodont amphibian *Dissorophus multicinctus*, and adaptations and phylogeny of the family Dissorophidae. J Paleontol 1968;42: 1210–1242.
14. Sigurdsen T, Bolt JR. The Lower Permian amphibamid *Doleserpeton* (Temnospondyli: Dissorophoidea), the interrelationships of amphibamids, and the origin of modern amphibians. J Vert Paleol. 2010;30: 1360–1377.
15. Berman DS, Reisz RR, Eberth DA. *Ecolsonia cutlerensis*, an Early Permian dissorophid amphibian from the Cutler Formation of north-central New Mexico. New Mexico Bureau of Mines & Mineral Resources 1985;191: 1-31.
16. Boy JA. Über die Micromelerpetontidae (Amphibia: Temnospondyli). 3. *Eimerisaurus* n. g.: Neues Jahrbuch für Geologie und Paläontologie, Abhandlungen. 2002;225: 425–452.
17. Jenkins FA Jr, Walsh DM, Carroll RL. Anatomy of *Eocaecilia micropodia*, a limbed caecilian of the Early Jurassic. Bull Mus Comp Zool. 2007;158: 285–365.
18. Daly E. Amphibamidae (Amphibia: Temnospondyli), with a description of a new genus from the upper Pennsylvanian of Kansas. Monogr Mus Nat Hist Univ Kans. 1994;85: 1-59.
19. Werneburg R, Lucas SG, Schneider JW, Rinehart LF. First Pennsylvanian *Eryops* (Temnospondyli) and its Permian record from New Mexico. Bull N M Mus Nat Hist Sci. 2010;49: 129–135.
20. Dempster WT. The braincase and endocranial cast of *Eryops megacephalus* (Cope). J Comp Neurol. 1935;62: 171–196.
21. Berman DS, Henrici AC, Brezinski DK, Kollar AD. A new trematopid amphibian (Temnospondyli: Dissorophoidea) from the Upper Pennsylvanian of western Pennsylvania: earliest record of terrestrial vertebrates responding to a warmer, drier climate. Ann Carnegie. 2009;78: 289–318.
22. Anderson JS, Henrici AC, Sumida SS, Martens T, Berman DS. *Georgenthalia clavinasica*, a new genus and species of dissorophoid temnospondyl from the Early Permian of Germany, and the relationships of the family Amphibamidae. J Vert Paleontol. 2008;28: 61–75.
23. Anderson JS, Reisz RR, Scott D, Fröbisch NB, Sumida SS. A stem batrachian from the Early Permian of Texas and the origin of frogs and salamanders. Nature. 2008;453: 515–518.
24. Schoch RR. Studies on braincases of early tetrapods: Structure, morphological diversity, and phylogeny-2. Dissorophoids, eryopids, and stereospondyls. Neues Jahrbuch für Geologie und Paläontologie - Abhandlungen 1999;213: 289– 312.
25. Ivachnenko MF. Urodelans from the Triassic and Jurassic of Soviet central Asia. Paleontol J. 1978;12: 362-368.
26. Boy JA. Studien über die Branchiosauridae (Amphibia: Temnospondyli) 1. Neue und wenig bekannte Arten aus dem mitteleuropäischen Rotliegenden (? oberstes Karbon bis unteres Perm). Paläontologische Zeitschrift 1986;60: 131–166.
27. Schoch RR. First evidence of the branchiosaurid temnospondyl *Leptorophus* in the Early Permian of the Saar-Nahe Basin (SW Germany). Neues Jahrbuch für Geologie und Paläontologie - Abhandlungen 2014;272: 225–236.
28. Werneburg, R. Dissorophoiden (Amphibia, Rhachitomi) aus dem Westfal D (Oberkarbon) von Böhmen. *Limnogyrinus elegans* (Fritsch 1881): Zeitschrift für geologische Wissenschaften. 1994;22: 457–467.
29. Boy JA. Über die Micromelerpetontidae (Amphibia: Temnospondyli). 1. Morphologie und Paläoökologie des *Micromelerpeton credneri* (Unter-Perm; SW-Deutschland). Paläontologische Zeitschrift. 1995;69: 429–457.
30. Schoch RR, Rubidge BS. The amphibamid *Micropholis* from the Lystrosaurus assemblage zone of South Africa. J Vert Paleontol. 2005;25: 502–522.
31. Ruta M, Bolt JR. A reassessment of the temnospondyl amphibian *Perryella olsoni* from the Lower Permian of Oklahoma. Earth Environ Sci Trans R Soc Edinb. 2006;97: 113–65.
32. Dilkes DW. A new trematopsid amphibian (Temnospondyli: Dissorophoidea) from the Lower Permian of Texas. J Vert Paleontol. 1990;10: 222–243.
33. Berman DS, Reisz RR, Fracasso MA. Skull of the Lower Permian dissorophid amphibian *Platyhystrix rugosus*. Ann Carnegie 1981;50: 391-416.
34. Clack JA, Milner AR. Morphology and systematics of the Pennsylvanian amphibian *Platyrhinops lyelli* (Amphibia: Temnospondyli). Earth Env Sci Trans R Soc. 2009;100: 275–295.
35. Huttenlocker AK, Pardo JD, Small BJ. *Plemmyradytes shintoni*, gen. et sp. nov., an Early Permian amphibamid (Temnospondyli: Dissorophoidea) from the Eskridge Formation, Nebraska. J Vert Paleontol. 2007;27: 316–328.
36. Maddin HC, Fröbisch NB, Evans DC, Milner AR. Reappraisal of the Early Permian amphibamid *Tersomius texensis* and some referred material. CR Palevol. 2013;12: 447–461.
37. Bourget H, Anderson JS. A new amphibamid (Temnospondyli: Dissorophoidea) from the Early Permian of Texas. J Vert Paleontol. 2011;31: 32–49.
38. Schoch RR, Witzmann F. Osteology and relationships of the temnospondyl genus *Sclerocephalus*. Zool J Linnean Soc. 2009;157: 135–168.
39. Sumida SS, Berman DS, and Martens T. A new trematopid amphibian from the Lower Permian of central Germany. Palaeontol. 1998;41: 605–630.
40. Anderson JS, Bolt JR. New information on amphibamids (Tetrapoda, Temnospondyli) from Richards Spur (Fort Sill), Oklahoma. J Vert Paleontol. 2013;33: 553–567.
41. Ascarrunz E, Rage J-C, Legreneur P, Laurin M. *Triadobatrachus massinoti*, the earliest known lissamphibian (Vertebrata: Tetrapoda) re-examined by μCT scan, and the evolution of trunk length in batrachians. Contrib Zool. 2016;85: 201–234.
42. Rage JC, and Rocek Z. Redescription of *Triadobatrachus massinoti* (Piveteau, 1936) an anuran amphibian from the early Triassic. Palaeontographica 1989;206: 1–16.
43. Milner AR, Schoch RR. *Trimerorhachis* (Amphibia: Temnospondyli) from the Lower Permian of Texas and New Mexico: Cranial osteology, taxonomy, and biostratigraphy: Neues Jahrbuch für Geologie und Paläontologie Abhandlungen. 2013;270: 91–128.
